# Effective tricuspid regurgitation reduction is associated with renal improvement and reduced heart failure hospitalization

**DOI:** 10.3389/fcvm.2024.1452446

**Published:** 2024-10-21

**Authors:** Dominik Felbel, Juliana von Winkler, Michael Paukovitsch, Matthias Gröger, Elene Walther, Stefanie Andreß, Leonhard Schneider, Sinisa Markovic, Wolfgang Rottbauer, Mirjam Keßler

**Affiliations:** Department of Cardiology, Ulm University Heart Center, Ulm, Germany

**Keywords:** tricuspid regurgitation, renal improvement, heart failure hospitalization, edge-to-edge repair, T-TEER

## Abstract

**Background:**

Several studies have demonstrated an association between tricuspid regurgitation (TR) and organ dysfunction including hepatic and renal insufficiency. Improvement of liver function following transcatheter edge-to-edge repair (T-TEER) has already been linked to reduction of venous congestion due to TR reduction. This study analyzes whether TR-reduction using T-TEER is also associated with improved renal function.

**Methods and results:**

The TRIC-ULM registry includes 92 selected patients undergoing T-TEER between March 2017 and May 2023. Estimated glomerular filtration rate (eGFR) improvement was evident in 53 patients (57%) at 3-months follow-up (FU) and defined by FU eGFR > baseline eGFR. Median age was 80 [interquartile range 75–83] years, pre- and postinterventional TR grades were 4 [3–5] and 1 [1–2], baseline eGFR was 36 [30–53] ml/min and New Yeark Heart Association (NYHA) IV was evident in 15% of patients. Multiple logistic regression analysis revealed TR vena contracta reduction (Odds ratio (OR) 1.35 [95% CI: 1.12–1.64] per mm, *p* = 0.002) and reduced preinterventional tricuspid annular plane systolic excursion (TAPSE) [OR 0.89 (95% CI: 0.79–0.99) per mm, *p* = 0.033] to independently predict renal improvement at FU. An eGFR improvement threshold of >9 ml/min was associated with reduced 1-year heart failure hospitalization rates [adjusted hazard ratio 0.22 (95% CI: 0.07–0.62) *p* = 0.005].

**Conclusion:**

Effective tricuspid edge-to-edge repair is associated with improved renal function and reduced heart failure hospitalization. In patients without renal improvement at 3-months follow-up, residual tricuspid regurgitation should be reevaluated for reintervention.

## Introduction

The TRILUMINATE trial demonstrated that symptomatic tricuspid regurgitation (TR) can successfully be treated using transcatheter edge-to-edge repair (T-TEER) (1). An association between atrioventricular valve regurgitation and hepatorenal function has already been reported and is assumed to be mediated by elevated venous congestion and reduced cardiac output (2–5).

The combination of reduced renal perfusion and elevated congestion is described as “renal tamponade” by Boorsma et al. in patients with heart failure and chronic kidney disease (6). Regarding mitral valve regurgitation (MR), large studies observed renal improvement in patients undergoing mitral valve edge-to-edge repair (M-TEER) (7). Regarding tricuspid valve regurgitation, Tanaka et al. found that an elevated model for end-stage liver disease excluding international normalized ratio (MELD-XI) score—reflecting hepatorenal function—predicts 1-year all-cause mortality and heart failure hospitalization (HFH) in patients who underwent T-TEER (8). Simultaneously, reducing TR to less than grade 3+ was independently associated with a reduction in MELD-XI score (8). Furthermore, postprocedural acute kidney injury has been linked to worse clinical outcomes following T-TEER (9). To further investigate the specific impact of T-TEER on renal function, our study aimed to investigate whether the effective reduction of tricuspid regurgitation is associated with improvement of renal function.

## Methods

### Study design and cohort

This retrospective single-center study included 92 consecutive patients undergoing T-TEER for symptomatic tricuspid regurgitation with 3 months available estimated glomerular filtration rate (eGFR) FU at our Ulm University Heart Center between March 2017 and May 2023. Patients undergoing reinterventions or patients on dialysis were excluded. Patients suffered from symptomatic heart failure equivalent to New York Heart Association (NYHA) ≥2 despite guideline-directed medical therapy. Baseline and postinterventional TR were defined according to the 2017 European Society of Cardiology (ESC) guidelines by intraprocedural transesophageal echocardiography (TEE) (10). eGFR was estimated using the Cockcroft-Gault-Formula. Each patient was evaluated by the interdisciplinary heart team according to current guidelines at the timepoint of procedure. All patients underwent T-TEER using the MitraClip (generation G1 to G3,) or TriClip (both Abbott Vascular) or PASCAL (PASCAL (P10) and PASCAL Ace (P5), Edwards Lifesciences). The objective of this study was to assess the impact of T-TEER on renal function. Therefore, eGFR improvement at 3 months following T-TEER was defined as primary endpoint. The closest eGFR value to 3 months follow-up was chosen within the time range 1 to 8 months after the T-TEER procedure. eGFR improvement was defined as follow-up eGFR > baseline eGFR considering that an eGFR slope of 0.75 ml/min/1.73 m^2^/year already impacts clinical outcomes as demonstrated by Odler et al. (11). One-year HFH was defined as secondary endpoint. Loop diuretic dosage was assessed in accordance with the established conversion standard: 20 mg torasemide = 40 mg furosemide oral administration. The investigation conforms with the principles outlined in the Declaration of Helsinki and was approved by the Ulm University ethics committee (435/16). Informed consent was obtained from all patients.

### Follow up

Follow-up was performed by routine clinical visit, by telephone or hospitalization at our Heart Center.

### Reference cohort

To demonstrate homogeneity, key baseline and procedure characteristics of the study cohort were compared to the excluded patients without follow-up. Male sex (57.6% in the included vs. 38.7% in the non-included group; *p* = 0.004) and higher MR grade (*p* < 0.001) were significantly more often and baseline creatinine (Median 140 [interquartile range 103–173] vs. 121 [97–154] µmol/L; *p* = 0.037) higher in patients with follow-up (FU). Left ventricular ejection fraction (LVEF) was lower (48 [38–57] vs. 50 [44–58]; *p* = 0.026) and postprocedural TR grade higher (*p* = 0.007) in the included cohort; Supplementary Table S1.

### Statistical analysis

The study cohort was grouped according to 3-month eGFR improvement and non-improvement. Data are displayed as mean ± standard deviation, median with 25th–75th percentiles, proportions (%) or Kaplan–Meier estimates (%). The distribution of continuous variables was tested with the Kolmogorov–Smirnov test. Normally distributed variables were analyzed using the student's *t*-test and non-normally distributed variables using the unpaired *U*-test. Categorial variables were compared by the chi-square test. All variables at baseline and the procedure results were dichotomized according to eGFR improvement. All variables were further analyzed by univariate logistic regression analysis (Supplementary Table S2). Pearson's and Spearman Rho correlation functions were used to identify relevant correlation (*r* > 0.4) between variables. Multiple logistic regression analysis (full model) to predict eGFR improvement included all significantly tested pre- and periprocedural variables of univariate logistic regression analysis. The strength of the association with eGFR improvement was estimated by the adjusted odds ratio (OR) and the 95% confidence interval. Multiple Cox regression analysis was calculated using the backwards likelihood ratio and included the following significant variables from univariate Cox regression analysis: age, invasive sPAP (mmHg), NYHA grade, TR grade reduction and renal improvement >9 (ml/min) and was estimated by the adjusted Hazard ratio (HR) and the 95%-confidence interval (Supplementary Table S3). A receiver operating characteristic (ROC) analysis estimating sensitivity, specificity and area under the curve (AUC) was performed and the optimal cut-off was identified by Youden's Index. Statistical analysis was performed using IBM® SPSS® Statistics Version 28.

## Results

### Study cohort

92 patients undergoing T-TEER with 3-month [93 (65–117) days] eGFR follow-up were included. Three-month eGFR improvement was evident in 53 (57.6%) patients.

Baseline characteristics such as age (79 [75–83] in the improved vs. 80 [74–83] years) in the non-improved cohort; *p* = 0.74), male sex (*p* = 0.84) and MR severity (*p* = 0.28) did not significantly differ between patients. Beside betablocker intake (91% vs. 74%; *p* = 0.038), heart failure medication and diuretic intake at discharge were comparable. Baseline eGFR was significantly lower in the improved cohort (34 [29–43] vs. 41 [31–63] ml/min; *p* = 0.025). eGFR at follow-up was found to be 45 [34–55] ml/min at median. Hemodynamic parameters were comparable. Pre- and postinterventional tricuspid regurgitation grades did not show a significant difference (*p* = 0.16 and *p* = 0.65), whereas TR vena contract reduction was higher in the improved cohort [8 (5–10)] vs. 4 [3–6] mm; *p* < 0.001) (Table 1).

**Table 1:** Baseline and procedural characteristics and outcome in patients undergoing T-TEER stratified by improvement of renal function during mid-term follow up.

	No renal improvement (*n* = 39)	Renal improvement (*n* = 53)	*P*-value
Baseline characteristics			
Age, years	80 [74–83] (39/39)	79 [75–83] (53/53)	0.742
Height, cm	168 ± 10 (39/39)	168 ± 10 (53/53)	0.793
Weight, kg	78 [63–93] (39/39)	75 [67–82] (53/53)	0.279
Male	22 (56.4%) (39/39)	31 (58.5%) (53/53)	0.842
Arterial hypertension	33 (84.6%) (39/39)	46 (86.8%) (53/53)	0.767
Hyperlipoproteinemia	27 (69.2%) (39/39)	37 (69.8%) (53/53)	0.952
Diabetes	14 (35.9%) (39/39)	16 (30.2%) (53/53)	0.546
Smoker	8 (20.5%) (39/39)	10 (18.9%) (53/53)	0.844
Creatinine, µmol/L	124 [81–170] (39/39)	147 [122–176] (53/53)	**0.041**
eGFR, ml/min	41 [31–63] (39/39)	34 [29–43] (53/53)	**0**.**025**
Baseline NYHA grade			0.226
II	8 (20.5%)	9 (17.0%)	
III	28 (71.8%)	33 (62.3%)	
IV	3 (7.7%) (39/39)	11 (20.8%) (53/53)	
Coronary artery disease			0.469
None	16 (41.0%)	25 (47.2%)	
1-vessel	9 (23.1%)	8 (15.1%)	
2-vessel	4 (10.3%)	10 (18.9%)	
3-vessel	10 (25.6%) (39/39)	10 (18.9%) (53/53)	
Dilatative cardiomyopathy	4 (10.3%) (39/39)	10 (17.0%) (53/53)	0.360
Pulmonary hypertension	19 (48.7%) (39/39)	31 (58.5%) (53/53)	0.352
Atrial fibrillation	34 (87.2%) (39/39)	47 (88.7%) (53/53)	0.827
COPD	3 (7.7%) (39/39)	4 (7.5%) (53/53)	0.979
EuroSCORE II	5.5 [2.9–8.9] (39/39)	6.5 [4.6–11.2] (53/53)	0.120
Prior mitral valve intervention	19 (48.7%) (39/39)	25 (47.2%) (53/53)	0.883
ACE-inhibitor	14 (35.9%) (39/39)	17 (32.1%) (53/53)	0.702
ARB	10 (25.6%) (39/39)	16 (30.2%) (53/53)	0.632
Betablocker	29 (74.4%) (39/39)	48 (90.6%) (53/53)	**0**.**038**
SGLT2-inhibitor	7 (17.9%) (39/39)	16 (30.2%) (53/53)	0.180
Aldosterone antagonist	20 (51.3%) (39/39)	32 (60.4%) (53/53)	0.384
Loop diuretics	37 (94.9%) (39/39)	49 (92.5%) (53/53)	0.642
Baseline loop diuretic dosage, mg	20 [10–40]	20 [10–40]	0.266
Thiazid diuretics	7 (17.9%) (39/39)	9 (17.0%) (53/53)	0.904
Right atrial pressure, mmHg	18 ± 5 (32/39)	17 ± 6 (43/53)	0.334
mPAP, mmHg	33 [25–39] (32/39)	31 [25–39] (47/53)	0.893
Invasive sPAP, mmHg	51 [38–58] (34/39)	50 [39–59] (47/53)	0.985
PCWP, mmHg	22 [17–28] (29/39)	20 [15–28] (44/53)	0.502
TR Etiology			0.209
Functional	32 (82.1%)	49 (92.5%)	
Degenerative	2 (5.1%)	0	
Mixed	4 (10.3%)	4 (7.5%)	
PM-induced	1 (2.6%) (39/39)	0 (53/53)	
TR grade			0.166
3	15 (38.5%)	14 (26.9%)	
4	17 (43.6%)	19 (36.5%)	
5	7 (17.9%) (39/39)	21 (36.5%) (52/53)	
TR vena contracta, mm	11 [8–12] (38/39)	13 [10–16] (53/53)	**0**.**005**
LVEF, %	46.1 ± 12.8% (36/39)	46.9 ± 14.2% (50/53)	0.752
TAPSE, mm	19 [15–23] (34/39)	18 [13–20] (48/53)	**0**.**034**
MR severity			0.283
Mild	27 (69.2%)	29 (54.7%)	
Moderate	8 (20.5%)	16 (30.2%)	
Severe	0 (39/39)	3 (5.7%) (53/53)	
Vena cava inferior, mm			
Preinterventional	23 [21–28] (37/39)	23 [20–30] (48/53)	0.127
3-months FU	22 [18–26] (33/39)	20 [17–27] (48/53)	0.893
Echocardiographic sPAP, mmHg	50 [35–65] (38/39)	50 [39–62] (51/53)	0.836
Procedural data and in-hospital outcomes			
Procedure duration, minutes	111 [77–143] (39/39)	100 [74–141] (52/53)	0.639
Fluoroscopy time, minutes	33 [21–44] (31/39)	31 [18–43] (45/53)	0.428
Device number			0.799
1	14 (35.9%)	20 (37.7%)	
2	22 (56.4%)	27 (50.9%)	
3	3 (7.7%) (39/39)	6 (11.3%) (53/53)	
Postinterventional TR grade			0.655
1	19 (48.7%)	27 (51.9%)	
2	10 (25.6%)	15 (28.8%)	
3	10 (25.6%)	9 (17.3%)	
4	0 (39/39)	1 (1.9%) (52/53)	
TR grade reduction			0.096
0	1 (2.6%)	1 (2.0%)	
1	6 (15.4%)	3 (5.9%)	
2	26 (66.7%)	26 (51.0%)	
3	5 (12.8%)	17 (33.3%)	
4	1 (2.6%) (39/39)	4 (7.8%) (51/53)	
Postinterventional TV mean gradient, mmHg	2 [1–3] (27/39)	2 [1–3] (45/53)	0.363
Time in-hospital, days	7 [6–10] (39/39)	7 [6–12] (53/53)	0.194
Postprocedural acute kidney injury	9 (23.1%) (39/39)	9 (17.0%) (53/53)	0.466
Postprocedural prolonged respiratoric weaning	1 (2.6%) (39/39)	4 (7.5%) (53/53)	0.297
Access site complication	1 (2.6%) (39/39)	2 (3.8%) (53/53)	0.747
Relevant pericardial effusion	0	0	–
Postprocedural need for catecholamines	2 (5.1%) (39/39)	6 (11.3%) (53/53)	0.298
Infection	2 (5.1%)	6 (11.3%)	0.298
Creatinine at discharge, µmol/L	117 [85–159] (39/39)	127 [97–152] (53/53)	0.978
eGFR at discharge, ml/min	45 [27–60] (39/39)	44 [31–57] (53/53)	0.959
TR vena contracta, mm			
Postinterventional	6 [4–8] (38/39)	5 [3–7] (52/53)	0.072
Vena contracta reduction	4 [3–6] (38/39)	8 [5–10] (52/53)	**<0.001**
3-month follow-up			
TR grade 3-month follow-up			0.829
0	1 (2.7%)	1 (2.3%)	
1	19 (51.4%)	22 (50.0%)	
2	9 (24.3%)	8 (18.2%)	
3	8 (21.6%)	12 (27.3%)	
4	0 (37/39)	1 (2.3%) (44/53)	
Creatinine at 3-month follow-up, µmol/L	152 [100–242] (39/39)	119 [101–145] (53/53)	0.013
eGFR at 3-month follow-up, ml/min	31 [20–50] (39/39)	45 [34–55] (53/53)	**<0**.**001**
Difference eGFR follow-up and baseline	−8 [−13 to (−5)] (39/39)	9 [4–15] (53/53)	**<0**.**001**
Vena cava inferior, mm			
3-month FU	22 [18–26] (33/39)	20 [17–27] (48/53)	0.893
Difference to baseline	−3 [(−8) to 4] (31/39)	−2 [(−5) to 1] (45/53)	0.217
NYHA grade at 3-month follow-up			0.127
1	5 (17.9%)	11 (28.9%)	
2	13 (46.4%)	16 (42.1%)	
3	10 (35.7%)	7 (18.4%)	
4	0 (28/39)	4 (10.5%) (38/53)	
One-year outcomes			
1-year heart failure hospitalization	18 (46.2%) (39/39)	23 (43.4%) (53/53)	0.793
1-year death	6 (15.4%) (39/39)	5 (9.4%) (53/53)	0.385

ARB, angiotensin receptor blocker; COPD, chronic obstructive pulmonary disease; eGFR, estimated glomerular filtration rate; FU, follow-up; PCWP, pulmonary capillary wedge pressure; LVEF, left ventricular ejection fraction; s-/mPAP, systolic-/mean pulmonary artery pressure; SGLT2, sodium glucose transport protein 2; NYHA, New York Heart Association; TAPSE, tricuspid annular plane systolic excursion; TR, tricuspid regurgitation; T-TEER, transcatheter edge-to-edge-repair.

*P*-values <0.05 are displayed bold and variable availability is displayed in brackets.

### Predictors of renal improvement at 3-month follow-up

**Figure 1:**
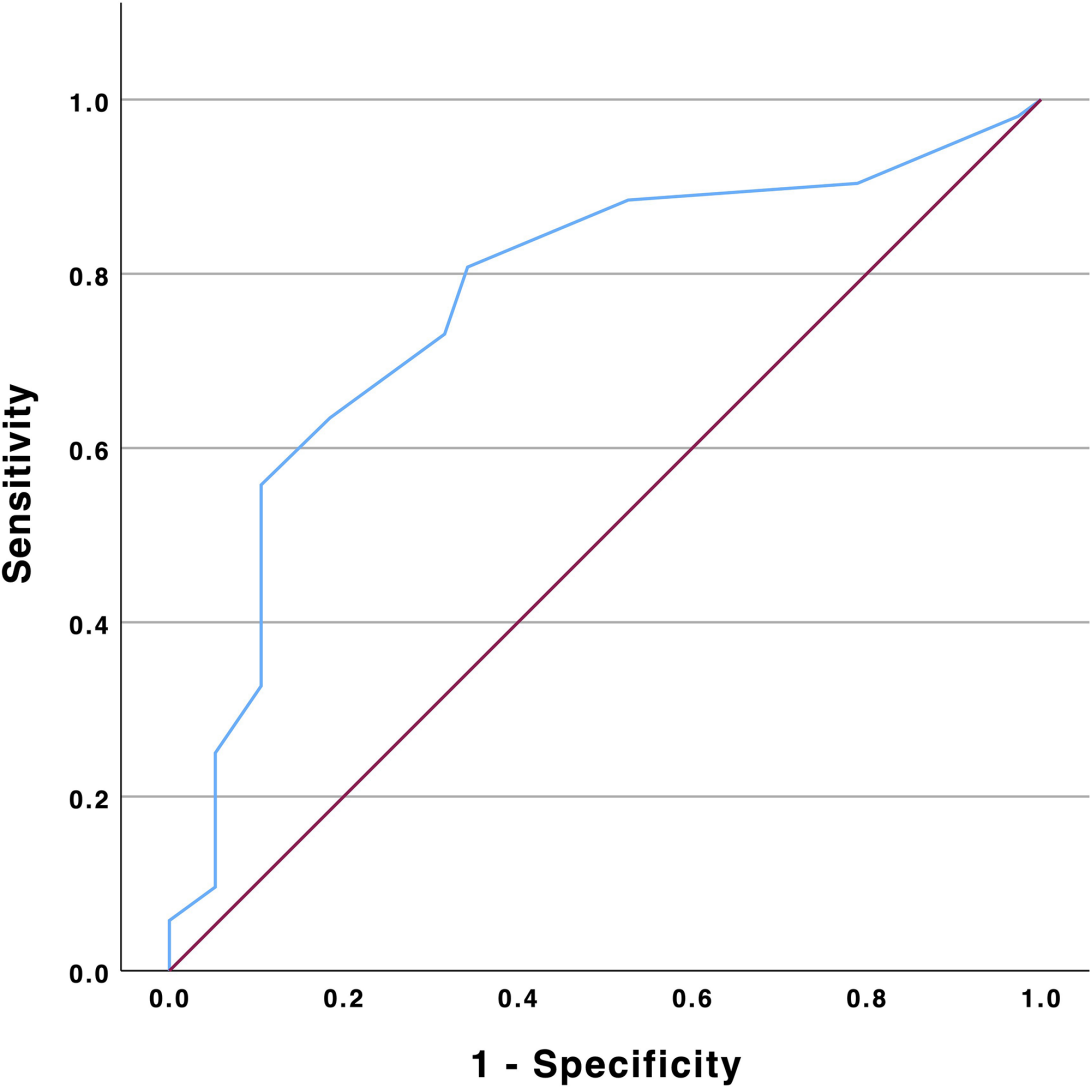
ROC analysis of TR vena contracta reduction and eGFR improvement. eGFR, estimated glomerular filtration rate; TR, tricuspid regurgitation.

Multiple logistic regression analysis revealed TR vena contracta reduction (VC) [HR 1.35 (95% CI: 1.12–1.64); *p* = 0.002] and tricuspid annular plane systolic excursion (TAPSE) [OR 0.89 (0.79–0.99); *p* = 0.033] to independently impact renal improvement at 3-month follow-up (Table 2). ROC analysis identified a threshold of 6.5 mm TR VC reduction to predict eGFR improvement at follow-up with a sensitivity of 64% and 82% specificity (AUC 0.77, 95% CI: 0.67–0.87; *p* < 0.001, Figure 1).

**Table 2:** Logistic regression analysis to identify predictors for renal function improvement during mid-term follow-up.

	Odds ratio	95%-Confidence interval	*P*-value
Univariate regression analysis			
Preinterventional betablocker	3.31	1.03–10.65	**0.045**
Baseline eGFR, ml/min	0.97	0.95–0.99	**0.030**
Preinterventional TAPSE, mm	0.89	0.82–0.98	**0.018**
Vena contracta reduction, mm	1.39	1.17–1.65	**<0.001**
Multiple regression analysis			
TR vena contracta reduction, mm	1.35	1.12–1.64	**0.002**
Preinterventional TAPSE, mm	0.89	0.79–0.99	**0.033**
Baseline eGFR, ml/min	1.00	0.97–1.03	0.890
Preinterventional betablocker	2.87	0.66–12.53	0.161

eGFR, estimated glomerular filtration rate; TAPSE, tricuspid annular plane systolic excursion; TR, tricuspid regurgitation.

*P*-values <0.05 are presented bold.

### Heart failure hospitalization

HFH occurred in 41 (44.6%) patients. These patients suffered more often from NYHA class IV (24% in rehospitalized vs. 8% in non-rehospitalized patients; *p* = 0.028), had an elevated invasive sPAP (55 ± 15 vs. 46 ± 14 mmHg; *p* = 0.007) and a lower baseline eGFR (32 [27–42] vs. 39 [32–63] ml/min; *p* = 0.023) (Supplementary Table S4). In patients with improved eGFR, ROC analysis identified a threshold of eGFR increase by 9 ml/min to predict a reduced HFH risk with a sensitivity of 67% and 78% specificity (AUC 0.72, 95% CI: 0.58–0.86; *p* = 0.006; Supplementary Figure S1). Patients with eGFR improvement >9 ml/min had a 1-year rehospitalization probability of 26.6%, whereas no improvement <9 ml/min was associated with a risk of 61.8% (Log rank *p* = 0.013). Multiple Cox logistic regression analysis revealed NYHA IV [HR 12.24 (95% CI: 3.15–47.53); *p* < 0.001] and eGFR improvement >9 ml/min [0.22 (95% CI: 0.07–0.62), *p* = 0.005] to independently impact HFH (Table 3).

**Table 3:** Cox regression analysis to identify predictors for unplanned 1-year heart failure hospitalization.

	Hazard ratio	95%-Confidence interval	*P*-value
Univariate Cox regression analysis			
Age, years	1.07	1.01–1.13	**0.019**
Invasive sPAP, mmHg	1.02	1.00–1.05	**0.031**
Baseline eGFR, ml/min	0.97	0.95–0.99	**0.004**
Preinterventional echocardiographic sPAP, mmHg	1.02	1.00–1.03	**0.016**
Baseline NYHA grade			
II			
III	3.38	1.02–11.18	**0.046**
IV	12.47	3.36–46.33	**<0.001**
Pulmonary hypertension	2.20	1.15–4.22	**0.017**
TR grade reduction			
0			
1	0.08	0.08–0.83	**0.035**
2	0.16	0.02–1.26	0.082
3	0.12	0.15–1.02	0.052
4	0.08	0.05–1.41	0.085
eGFR improvement >9 ml/min	0.27	0.09–0.76	**0.013**
Multiple Cox regression analysis			
Baseline NYHA grade			
II			
III	2.49	0.75–8.37	0.138
IV	12.24	3.15–47.53	**<0.001**
eGFR improvement >9 ml/min	0.22	0.07–0.62	**0.005**

eGFR, estimated glomerular filtration rate; sPAP, systolic pulmonary artery pressure; NYHA, New York Heart Association; TR, tricuspid regurgitation.

*P*-values <0.05 are displayed bold.

## Discussion

To date, this is the largest study to investigate the impact of T-TEER on renal function. The main results can be summarized as follows:

- Renal improvement during mid-term follow-up was evident in the majority of patients (57%)
- Tricuspid regurgitation reduction indicated by TR vena contracta reduction and lower preinterventional TAPSE indicating right ventricular dysfunction predict renal improvement during follow-up
- Significant renal improvement is associated with reduced heart failure hospitalization (adjusted HR 0.22)

Several studies demonstrated an association between tricuspid regurgitation and impaired renal function (4, 5). Reduced cardiac output and elevated venous congestion are both assumed to contribute to the negative effect of TR on renal function (3–5, 12). In patients with relevant TR undergoing T-TEER, renal function may improve by reducing tricuspid regurgitation. Our study confirms this assumption and demonstrates renal improvement in 57% of patients. Furthermore, TR predicts renal improvement emphasizing the procedures’ beneficial effect on renal function. Additionally, patients with reduced TAPSE had a higher chance of mid-term eGFR improvement. Thus, sicker patients seem to benefit even more from the effect of venous decongestion.

Wang et al. demonstrated both MR reduction and more advanced chronic kidney disease to predict renal improvement following the M-TEER procedure (7). Moreover, CKD stage correlated directly with worsened prognosis in their analysis (7). In our study, impaired preinterventional renal function showed a trend towards higher risk of heart failure rehospitalization (*p* = 0.08). However, in patients with a relevant eGFR improvement >9 ml/min at mid-term follow-up, the likelihood of heart failure rehospitalization was significantly reduced by 68% (95% CI: 18%–87%).

Renal improvement was observed in the majority of patients (57%). Notably, patients who experienced renal improvement had a higher preinterventional TR VC, and the reduction of TR VC was more in these patients. Each millimeter reduction of VC reduction was associated with a 35% increased likelihood of renal improvement (OR 1.35), highlighting the direct link between venous renal decongestion and a patients’ individual absolute TR reduction.

Additionally, a lower TAPSE was associated with a higher likelihood of renal improvement (OR 0.89). This finding aligns with recent research by Vogelhuber et al., which showed that TAPSE improved following T-TEER in patients with reduced preinterventional TAPSE (13). This oberservation underscores the previously described critical role of right ventricular function in influencing renal function and demonstrates a potentially benefit of T-TEER in this context (14).

However, renal improvement was not observed in every subject of the whole cohort, which may be explained by the following: chronic kidney disease itself displays a risk-factor of valvular heart disease mediated by volume overload causing annulus dilatation and degeneration by calcification and therefore, renal improvement seems unlikely in these patients (15). On the other hand, patients without preinterventional renal impairment are not expected to show renal improvement during follow-up.

Consequently, renal improvement may be used as a surrogate of the T-TEER procedures’ efficacy indicating improved organ function and being associated with reduced HFH in selected patients.

The observed HFH rate of 45% is higher than in other studies, such as the randomized TRILUMINATE study reporting 14.9% (1). This can be explained by the fact that only patients with follow-up eGFR were included in our study. Consequently, patients without need for short-term blood control or not experiencing heart failure rehospitalization are not included in our study cohort due to lacking eGFR control. When comparing the included and the not included cohort, LVEF was lower and mitral regurgitation severity was higher in the included cohort being both associated with elevated rehospitalization rates (16, 17). Hence, the results of our study especially focus on patients with a high risk of heart failure rehospitalization and demonstrates that optimal TR reduction is crucial in this vulnerable group.

## Limitations

The results of our study have to be interpreted with several confinements. The retrospective study design is associated with all the inherent limitations ascribed to this study type. Patients were dichotomized by renal improvement at mid-term follow-up irrespective of their CKD stage. Renal improvement was included to the Cox regression model for 1-year heart failure hospitalization, even though it was assessed at 3-month follow-up and not at baseline. However, it considers that complex hemodynamic changes have to apply and cannot be expected immediately after the procedure, underlining its value as an indicator of improved outcome.

## Conclusion

In patients with effective TR reduction and impaired renal function tricuspid edge-to-edge repair is associated with improvement of renal function. Renal improvement itself is associated with reduced heart failure hospitalization. In patients without renal improvement at 3-months follow-up, residual TR should be reevaluated.

## Data Availability

The datasets presented in this article are not readily available due to privacy reasons, but the raw data supporting the conclusions of this article will be made available on reasonable request from the corresponding author.

## Supplementary material

Supplementary Table S1 Baseline characteristics in patients undergoing T-TEER stratified by patients with available follow-up for eGFR.

Supplementary Table S2 Univariate logistic regression analysis to identify predictors for renal function improvement during mid-term follow-up.

Supplementary Table S3 Univariate Cox logistic regression analysis to identify predictors for unplanned 1-year heart failure hospitalization.

Supplementary Table S4 Baseline and procedural characteristics in patients undergoing T-TEER stratified by 1-year heart failure hospitalization.

Supplementary Figure S1 ROC analysis of eGFR improvement and heart failure hospitalization. eGFR, estimated glomerular filtration rate.
