# Supplementary material for: Effective tricuspid regurgitation reduction is associated with renal improvement and reduced heart failure hospitalization
Source: Front Cardiovasc Med. 2024 Oct 21;11:1452446. doi: 10.3389/fcvm.2024.1452446 (PMC11532059; doi:10.3389/fcvm.2024.1452446)
Supplement: Supplementary Table S4 — Baseline and procedural characteristics in patients undergoing T-TEER stratified by 1-year heart failure hospitalization. [file Table1.pdf]

Supplementary Material

**Supplementary Table S1** Baseline characteristics in patients undergoing T-TEER stratified by patients with available follow-up for eGFR			
	Patients without FU (n=163)	Patients with FU (n=92)	P-value
Age, years	80 [74 - 83] *(163/163)*	80 [75 - 83] *(92/92)*	0.686
Height, cm	168 [160 - 175] *(163/163)*	169 [160 - 178] *(92/92)*	0.598
Weight, kg	70 [62 - 84] *(163/163)*	75 [67 - 87] *(92/92)*	0.050
Male	63 (38.7%) *(163/163)*	53 (57.6%) *(92/92)*	**0.004**
Arterial hypertension	136 (83.4%) *(163/163)*	79 (85.9%) *(92/92)*	0.608
Hyperlipidemia	102 (62.6%) *(163/163)*	64 (69.6%) *(92/92)*	0.261
Diabetes	35 (21.5%) *(163/163)*	30 (32.6%) *(92/92)*	0.050
Smoker	22 (13.6%) *(162/163)*	18 (19.6%) *(92/92)*	0.208
Baseline NYHA grade<br>II<br>III<br>IV	 38 (22.7%)<br>106 (65.0%)<br>19 (11.7%)<br>*(163/163)*	 17 (18.5%)<br>61 (66.3%)<br>14 (15.2%)<br>*(92/92)*	0.648
Coronary artery disease<br>None<br>1-vessel<br>2-vessel<br>3-vessel	 84 (51.5%)<br>26 (16.0%)<br>22 (13.5%)<br>31 (19.0%)<br>*(163/163)*	 41 (44.6%)<br>17 (18.5%)<br>14 (15.2%)<br>20 (21.7%)<br>*(92/92)*	0.544
Dilatative cardiomyopathy	13 (8.0%) *(163/163)*	13 (14.1%) *(92/92)*	0.169
Cerebrovascular disease	29 (17.8%) *(163/163)*	9 (10.0%) *(92/92)*	0.097
Atrial fibrillation	148 (90.8%) *(163/163)*	81 (88.0%) *(92/92)*	0.485
COPD	20 (12.3%) *(163/163)*	7 (7.6%) *(92/92)*	0.438
EuroSCORE II	5.8 [3.4 – 9.6] *(160/163)*	6.2 [4.0 – 10.1] *(92/92)*	0.503
MR severity<br>None<br>Mild<br>Moderate<br>Severe	 52 (35.4%)<br>77 (52.4%)<br>12 (8.2%)<br>6 (4.1%)<br>*(147/163)*	 9 (9.8%)<br>56 (60.9%)<br>24 (26.1%)<br>3 (3.3%)<br>*(92/92)*	**<0.001**

Baseline creatinine, µmol/l	121 [97 - 154] *(163/163)*	140 [103 - 173] *(92/92)*	**0.037**
Baseline eGFR, ml/min	42 [29 - 56] *(163/163)*	36 [30 - 53] *(92/92)*	0.257
LVEF, %	50 [44 - 58] *(152/163)*	48 [38 - 57] *(86/92)*	**0.026**
**Procedural data**			
Procedure duration, minutes	100 [75 - 123] *(131/163)*	105 [75 - 143] *(91/92)*	0.524
TR grade			0.819
3	41 (28.1%)	29 (31.9%)	
4	60 (41.1%)	36 (39.6%)	
5	45 (30.8%) *(146/163)*	26 (28.6%) *(91/92)*	
TR grade post Clip			**0.007**
0	17 (10.6%)	0	
1	67 (41.9%)	46 (50.5%)	
2	43 (26.9%)	25 (27.5%)	
3	25 (15.6%)	19 (20.9%)	
4	8 (5.0%)	1 (1.1%)	
5	0 *(160/163)*	0 *(91/92)*	

P-values <0.05 are presented bold. Variable availability is displayed in brackets.
COPD: chronic obstructive pulmonary disease; eGFR: estimated glomerular filtration rate; LVEF: left ventricular ejection fraction; NYHA: New York Heart Association; TR: tricuspid regurgitation;T-TEER: transcatheter edge-to-edge-repair

Supplementary Table S2 Univariate logistic regression analysis to identify predictors for renal function improvement during mid-term follow-up

Univariate regression analysis			
	Odds ratio	95%-Confidence interval	P-value
Age, years	1.00	0.95 – 1.06	0.925
Height, cm	1.00	0.96 – 1.04	0.962
Weight, kg			
- Baseline	0.99	0.97 – 1.01	0.257
- 3-months FU	0.98	0.96 -1.01	0.144
- Difference	0.99	0.95 – 1.05	0.911
Male	0.92	0.39 – 2.12	0.842
Arterial hypertension	1.19	0.37 – 3.88	0.767
Hyperlipoproteinemia	1.03	0.42 – 2.52	0.952
Diabetes	0.63	0.26 – 1.52	0.306
Smoker	0.90	0.32 – 2.55	0.844
Baseline NYHA grade			
III	1.42	0.48 – 4.16	0.527
IV	4.13	0.84 – 20.28	0.081
NYHA IV	0.98	0.31 – 3.09	0.969

Coronary artery disease<br>1-vessel<br>2-vessel<br>3-vessel	 0.57<br>1.60<br>0.64	 0.18 – 1.78<br>0.43 – 5.98<br>0.22 – 1.88	 0.332<br>0.485<br>0.417
Dilatative cardiomyopathy	1.79	0.51 – 6.30	0.365
Pulmonary hypertension	1.48	0.65 – 3.41	0.353
Atrial fibrillation	1.15	0.33 – 4.09	0.827
COPD	0.98	0.21 – 4.65	0.979
EuroSCORE II	1.06	0.98 – 1.16	0.149
MR severity<br>Mild<br>Moderate<br>Severe	 0.92<br>1.33<br>-	 0.22 – 3.80<br>0.28 – 6.30<br>-	 0.912<br>0.717<br>-
Prior mitral valve intervention	0.79	0.34 – 1.80	0.569
ACE-inhibitor	0.69	0.29 – 1.65	0.408
ARB	1.57	0.61 – 4.04	0.345
Betablocker	3.31	1.03 – 10.65	**0.045**
SGLT2-inhibitor	1.53	0.57 – 4.08	0.396
Aldosterone antagonist	1.45	0.63 – 3.34	0.385
Loop diuretics	1.39	0.27 – 7.28	0.698
Loop diuretics dosage Admission	1.00	0.99 – 1.01	0.973
Thiazid diuretics	0.79	0.22 – 2.79	0.714
Baseline creatinine, µmol/l	1.00	0.99 – 1.01	0.368
Baseline eGFR, ml/min	0.97	0.95 – 0.99	**0.030**
Creatinine at discharge, µmol/l	0.99	0.99 – 1.00	0.339
eGFR at discharge, ml/min	1.00	0.98 – 1.02	0.743
Etiology<br>Functional/mixed<br>Degenerative<br>PM-induced	 -<br>-<br>-	 -<br>-<br>-	 0.99<br>0.99<br>1.00
LVEF, %	1.01	0.97 – 1.04	0.718
TAPSE, mm	0.89	0.82 – 0.98	**0.018**
Vena cava inferior, mm<br>- Preinterventional<br>- 3-months FU<br>- Difference	 1.02<br>1.03<br>1.01	 0.96 – 1.08<br>0.97 – 1.09<br>0.95 – 1.07	 0.592<br>0.360<br>0.783
Echocardiographic sPAP, mmHg	1.01	0.99 – 1.03	0.485
Procedure duration, minutes	0.99	0.99 – 1.01	0.739
Fluoroscopy time, minutes	0.99	0.97 – 1.01	0.405

Device number	1.05	0.54 – 2.02	0.892
Right atrial pressure, mmHg	0.96	0.88 – 1.04	0.291
mPAP, mmHg	1.01	0.96 – 1.06	0.646
Invasive sPAP, mmHg	1.01	0.98 – 1.04	0.649
PCWP, mmHg	0.99	0.93 – 1.05	0.657
Vena contracta, mm			
- Preinterventional	1.12	1.01 – 1.25	**0.036**
- Postinterventional	0.85	0.71 – 1.01	0.057
- Reduction	1.39	1.17 – 1.66	**<0.001**
TR grade			
3			
4	1.27	0.48 – 3.41	0.632
5	2.91	0.94 – 9.02	0.065
Postinterventional TR grade			
1			
2	1.06	0.39 – 2.85	0.915
3	0.63	0.22 – 1.86	0.405
4	-	-	1.0
TR grade reduction			
0			
1	2.0	0.09 – 44.35	0.661
2	1.36	0.08 – 23.01	0.830
3	1.44	0.08 – 26.23	0.804
4	0.67	0.03 – 18.06	0.810
Postinterventional TV mean gradient, mmHg	0.78	0.52 – 1.18	0.233
Time in-hospital, days	1.06	0.97 – 1.17	0.215
Postprocedural acute kidney injury	0.68	0.24 – 1.92	0.468
Postprocedural prolonged respiratoric weaning	3.10	0.33 – 28.90	0.320
Postprocedural need for catecholamines	2.36	0.45 – 12.39	0.309
Infection	2.36	0.45 – 12.39	0.309
TR grade at mid-term follow-up			
0			
1	1.05	0.06 – 17.95	0.973
2	1.13	0.06 – 21.09	0.937
3	1.50	0.08 – 27.61	0.785
4	-	-	1.0
NYHA grade at mid-term follow-up			
1			
2	0.36	0.09 – 1.37	0.134
3	0.23	0.05 – 1.03	0.055
4	-	-	0.99

P-values <0.05 are presented bold.
ARB: angiotensin receptor blocker; COPD: chronic obstructive pulmonary disease; eGFR: estimated glomerular filtration rate; FU: follow-up; PCWP: pulmonary capillary wedge pressure; LVEF: left ventricular ejection fraction; s-/mPAP: systolic-/mean pulmonary artery pressure; SGLT2: sodium glucose transport protein 2; NYHA: New York Heart Association; TAPSE: tricuspid annular plane systolic excursion; TR: tricuspid regurgitation; T-TEER: transcatheter edge-to-edge-repair

Supplementary Table S3 Univariate Cox logistic regression analysis to identify predictors for unplanned 1-year heart failure hospitalization

Univariate regression analysis			
	Hazard ratio	95%-Confidence interval	P-value
Age, years	1.07	1.01 – 1.13	**0.019**
Height, cm	0.98	0.95 – 1.02	0.305
Weight, kg			
- Baseline	0.99	0.97 – 1.01	0.277
- 3-months FU	0.99	0.97 – 1.01	0.173
- Difference	1.00	0.96 – 1.04	0.988
Male	0.75	0.41 – 1.39	0.370
Arterial hypertension	0.66	0.29 – 1.49	0.312
Hyperlipoproteinemia	0.64	0.34 – 1.20	0.163
Diabetes	1.06	0.55 – 2.06	0.854
Smoker	0.89	0.39 – 2.00	0.772
Baseline NYHA grade			
III	3.38	1.02 – 11.18	**0.046**
IV	12.47	3.36 – 46.33	**<0.001**
Coronary artery disease			
1-vessel	0.82	0.33 – 2.05	0.677
2-vessel	0.99	0.39 – 2.48	0.988
3-vessel	1.02	0.46 – 2.24	0.964
Dilatative cardiomyopathy	1.15	0.51 – 2.61	0.730
Pulmonary hypertension	2.20	1.15 – 4.22	**0.017**
Atrial fibrillation	1.11	0.44 – 2.83	0.828
COPD	0.25	0.03 – 1.81	0.170
EuroSCORE II	1.04	0.99 – 1.09	0.144
MR severity			
Mild	0.71	0.29 – 1.74	0.450
Moderate	0.67	0.24 – 1.83	0.432
Severe	0.88	0.18 – 4.37	0.875
Prior mitral valve intervention	1.73	0.92 – 3.24	0.088
ACE-inhibitor	0.51	0.24 – 1.06	0.071
ARB	1.22	0.63 – 2.36	0.549
Betablocker	3.04	0.94 – 9.85	0.064
SGLT2-inhibitor	1.18	0.56 – 2.49	0.658
Aldosterone antagonist	0.94	0.51 – 1.74	0.839
Loop diuretics	3.08	0.42 – 22.43	0.267

Loop diuretics dosage Admission	1.00	0.99 – 1.01	0.163
Thiazid diuretics	1.29	0.53 – 3.12	0.576
Baseline creatinine, µmol/l	1.004	1.000 – 1.009	0.066
Baseline eGFR, ml/min	0.97	0.95 – 0.99	**0.004**
Creatinine at discharge, µmol/l	1.01	1.00 – 1.01	**0.002**
eGFR at discharge, ml/min	0.97	0.95 – 0.99	**<0.001**
Creatinine at follow-up, µmol/l	1.004	1.001 – 1.007	**0.006**
eGFR at follow-up, ml/min	0.97	0.95 – 0.98	**<0.001**
Etiology<br>Functional/mixed<br>Degenerative<br>PM-induced	 22.53<br>0.05<br>0.05	 0.05 – 10699.72<br>0.00 – 82.61<br>0.00 – 1761.67	 0.322<br>0.421<br>0.571
LVEF, %	0.98	0.96 – 1.00	0.083
TAPSE, mm	0.99	0.93 – 1.05	0.709
Vena cava inferior, mm<br>- Preinterventional<br>- 3-months FU<br>- Difference	 1.03<br>1.01<br>0.99	 0.98 – 1.07<br>0.97 – 1.05<br>0.95 – 1.04	 0.235<br>0.591<br>0.734
Echocardiographic sPAP, mmHg	1.02	1.00 – 1.03	**0.016**
Procedure duration, minutes	1.03	0.99 – 1.07	0.076
Fluoroscopy time, minutes	1.001	0.986 – 1.016	0.912
Device number	0.87	0.53 – 1.42	0.568
Right atrial pressure, mmHg	1.01	0.95 – 1.07	0.881
mPAP, mmHg	1.02	0.99 – 1.06	0.197
Invasive sPAP, mmHg	1.02	1.00 – 1.05	**0.031**
PCWP, mmHg	1.01	0.97 – 1.06	0.681
Vena contracta, mm<br>- Preinterventional<br>- Postinterventional<br>- Reduction	 1.01<br>0.97<br>1.04	 0.95 – 1.08<br>0.86 – 1.09<br>0.95 – 1.13	 0.803<br>0.610<br>0.420
TR grade<br>3<br>4<br>5	 3.24<br>1.41<br>1.19	 0.41 – 25.59<br>0.64 – 3.08<br>0.52 – 2.77	 0.265<br>0.391<br>0.679
Postinterventional TR grade			

1			
2	1.87	0.92 – 3.79	0.082
3	1.16	0.49 – 2.73	0.741
4	2.19	0.29 – 16.68	0.448
TR grade reduction			
0			
1	0.08	0.08 – 0.83	**0.035**
2	0.16	0.02 – 1.26	0.082
3	0.12	0.15 – 1.02	0.052
4	0.08	0.05 – 1.41	0.085
Postinterventional TV mean gradient, mmHg	0.96	0.71 – 1.29	0.769
Time in-hospital, days	1.03	0.99 - 1.07	0.076
Postprocedural acute kidney injury	2.04	1.04 – 4.01	**0.039**
Postprocedural prolonged respiratoric weaning	3.19	1.13 – 9.06	**0.029**
Postprocedural need for catecholamines	2.28	0.89 – 5.84	0.086
Infection	1.17	0.42 – 3.28	0.770

P-values <0.05 are presented bold.
ARB: angiotensin receptor blocker; COPD: chronic obstructive pulmonary disease; eGFR: estimated glomerular filtration rate; FU: follow-up; PCWP: pulmonary capillary wedge pressure; LVEF: left ventricular ejection fraction; s-/mPAP: systolic-/mean pulmonary artery pressure; SGLT2: sodium glucose transport protein 2; NYHA: New York Heart Association; TAPSE: tricuspid annular plane systolic excursion; TR: tricuspid regurgitation;T-TEER: transcatheter edge-to-edge-repair

Supplementary Table S4 Baseline and procedural characteristics in patients undergoing T-TEER stratified by 1-year heart failure hospitalization

	No HF hospitalization (n=51)	HF Hospitalization (n=41)	P-value
Age, years	79 [75 - 81] (51/51)	80 [76 - 85] (41/41)	0.067
Height, cm	168 ±10 (51/51)	168 ±10 (41/41)	0.826
Weight, kg	80 [69 - 89] (51/51)	73 [64 – 83] (41/41)	0.290
Male	30 (58.8%) (51/51)	23 (56.1%) (41/41)	0.793
Arterial hypertension	45 (88.2%) (51/51)	34 (82.9%) (41/41)	0.468
Hyperlipoproteinemia	38 (74.5%) (51/51)	26 (63.4%) (41/41)	0.250
Diabetes	15 (29.4%) (51/51)	15 (36.6%) (41/41)	0.466
Smoker	11 (21.6%) (51/51)	7 (17.1%) (41/41)	0.589
Baseline NYHA			**0.010**
grade	14 (27.5%)	3 (7.3%)	
II	33 (64.7%)	28 (68.3%)	
III	4 (7.8%)	10 (24.4%)	

IV	(51/51)	(41/41)	
NYHA IV	4 (7.8%)<br>(51/51)	10 (24.4%)<br>(41/41)	**0.028**
Coronary artery disease<br>None<br>1-vessel<br>2-vessel<br>3-vessel	 21 (41.2%)<br>11 (21.6%)<br>8 (15.7%)<br>11 (21.6%)<br>(51/51)	 20 (48.8%)<br>6 (14.6%)<br>6 (14.6%)<br>9 (22.0%)<br>(41/41)	0.824
Dilatative cardiomyopathy	6 (11.8%)<br>(51/51)	7 (17.1%)<br>(41/41)	0.468
Pulmonary hypertension	24 (47.1%)<br>(51/51)	26 (63.4%)<br>(41/41)	0.117
Atrial fibrillation	45 (88.2%)<br>(51/51)	36 (87.8%)<br>(41/41)	0.950
COPD	6 (11.8%)<br>(51/51)	1 (2.4%)<br>(41/41)	0.094
EuroSCORE II	5.3 [3.2 – 10.1]<br>(51/51)	7.4 [5.2 – 10.3]<br>(41/41)	**0.042**
MR severity<br>None<br>Mild<br>Moderate<br>Severe	 3 (5.9%)<br>33 (64.7%)<br>14 (27.5%)<br>1 (2.0%)<br>(51/51)	 6 (14.6%)<br>23 (56.1%)<br>10 (24.4%)<br>2 (4.9%)<br>(41/41)	0.435
ACE-inhibitor	22 (43.1%)<br>(51/51)	9 (22.0%)<br>(41/41)	**0.033**
ARB	13 (25.5%)<br>(51/51)	13 (31.7%)<br>(41/41)	0.510
Betablocker	43 (80.4%)<br>(51/51)	36 (87.8%)<br>(41/41)	0.339
SGLT2-inhibitor	12 (23.5%)<br>(51/51)	11 (26.8%)<br>(41/41)	0.716
Aldosterone antagonist	30 (58.8%)<br>(51/51)	22 (53.7%)<br>(41/41)	0.619
Loop diuretics	46 (90.2%)<br>(51/51)	40 (97.6%)<br>(41/41)	0.155
Thiazid diuretics	7 (13.7%)<br>(51/51)	9 (22.0%)<br>(41/41)	0.301
Invasive sPAP, mmHg	46 ±14<br>(43/51)	55 ±15<br>(38/41)	**0.007**
PCWP, mmHg	20 ±7<br>(40/51)	23 ±7<br>(33/41)	0.140
Renal function			
Baseline creatinine, µmol/l	132 [88 - 157]<br>(51/51)	150 [119 - 186]<br>(41/41)	0.031
Baseline eGFR, ml/min	39 [32 - 63]<br>(51/51)	32 [27 - 42]<br>(41/41)	**0.008**
Creatinine at discharge, µmol/l	114 [80 - 141]<br>(51/51)	144 [102 - 196]<br>(41/41)	**0.007**
eGFR at discharge, ml/min	52 ±21<br>(51/51)	39 ±18<br>(41/41)	**0.002**
Creatinine at follow-up, µmol/l	113 [89 - 145]<br>(51/51)	151 [117 - 188]<br>(41/41)	**0.002**

eGFR at follow-up, ml/min	49 ±19<br>(51/51)	35 ±17<br>(41/41)	**<0.001**
Difference eGFR follow-up and baseline	3.4 ±13.4<br>(51/51)	-0.5 ±11<br>(41/41)	0.135
Echocardiographic data			
Etiology<br>Functional<br>Degenerative<br>Mixed<br>PM-induced	 45 (88.2%)<br>2 (3.9%)<br>3 (5.9%)<br>1 (2.0%)<br>(51/51)	 36 (87.8%)<br>0<br>5 (12.2%)<br>0<br>(41/41)	0.327
LVEF, %	49 ±14<br>(47/51)	43 ±13<br>(39/41)	**0.028**
Echocardiographic sPAP, mmHg	42 [35 - 60]<br>(49/51)	56 [44 - 69]<br>(40/41)	**0.007**
Procedural data			
Procedure duration, minutes	109 [75 - 152]<br>(50/51)	92 [75 - 121]<br>(41/41)	0.475
Fluoroscopy time, minutes	31 [19 - 43]<br>(39/51)	33 [21 - 43]<br>(37/41)	0.358
Tricuspid Vena contracta, cm	1.3 [1.0 – 1.5]<br>(40/51)	1.3 [0.9 – 1.5]<br>(38/41)	0.794
Device number<br>1<br>2<br>3	 18 (35.3%)<br>26 (51.0%)<br>7 (13.7%)<br>(51/51)	 16 (39.0%)<br>23 (56.1%)<br>2 (4.9%)<br>(41/41)	0.365
Right atrial pressure, mmHg	17 ±5<br>(39/51)	18 ±6<br>(36/41)	0.300
mPAP, mmHg	29 [25 - 38]<br>(42/51)	35 [27 - 40]<br>(37/41)	**0.024**
TR grade<br>3<br>4<br>5	 19 (37.3%)<br>18 (35.3%)<br>14 (27.5%)<br>(51/51)	 10 (25.0%)<br>18 (45.0%)<br>12 (30.0%)<br>(40/41)	0.443
Postinterventional TR grade<br>1<br>2<br>3<br>4	 <br>31 (60.8%)<br>9 (17.6%)<br>13 (21.6%)<br>0<br>(51/51)	 <br>15 (37.5%)<br>16 (40.0%)<br>8 (20.0%)<br>1 (2.5%)<br>(40/41)	0.051
TR grade reduction<br>0<br>1<br>2<br>3<br>4	 1 (2.0%)<br>6 (11.8%)<br>27 (52.9%)<br>13 (25.5%)<br>4 (7.8%)<br>(51/51)	 1 (2.6%)<br>3 (7.7%)<br>25 (64.1%)<br>9 (23.1%)<br>1 (2.6%)<br>(39/41)	0.728

TR grade reduction ≤3	17 (33.3%)<br>(51/51)	10 (25.6%)<br>(39/41)	0.430
Postinterventional TV mean gradient, mmHg	2 [1 - 3]<br>(40/51)	2 [1 - 3]<br>(32/41)	0.995
Time in-hospital, days	7 [6 - 10]<br>(51/51)	7 [6 - 12]<br>(41/41)	0.704

P-values <0.05 are displayed bold and variable availability is displayed in brackets.
ARB: angiotensin receptor blocker; COPD: chronic obstructive pulmonary disease; eGFR: estimated glomerular filtration rate; FU: follow-up; PCWP: pulmonary capillary wedge pressure; LVEF: left ventricular ejection fraction; s-/mPAP: systolic-/mean pulmonary artery pressure; SGLT2: sodium glucose transport protein 2; NYHA: New York Heart Association; TAPSE: tricuspid annular plane systolic excursion; TR: tricuspid regurgitation;T-TEER: transcatheter edge-to-edge-repair
