# Supplementary figures and images for: Effective tricuspid regurgitation reduction is associated with renal improvement and reduced heart failure hospitalization
Source: Front Cardiovasc Med. 2024 Oct 21;11:1452446. doi: 10.3389/fcvm.2024.1452446 (PMC11532059; doi:10.3389/fcvm.2024.1452446)

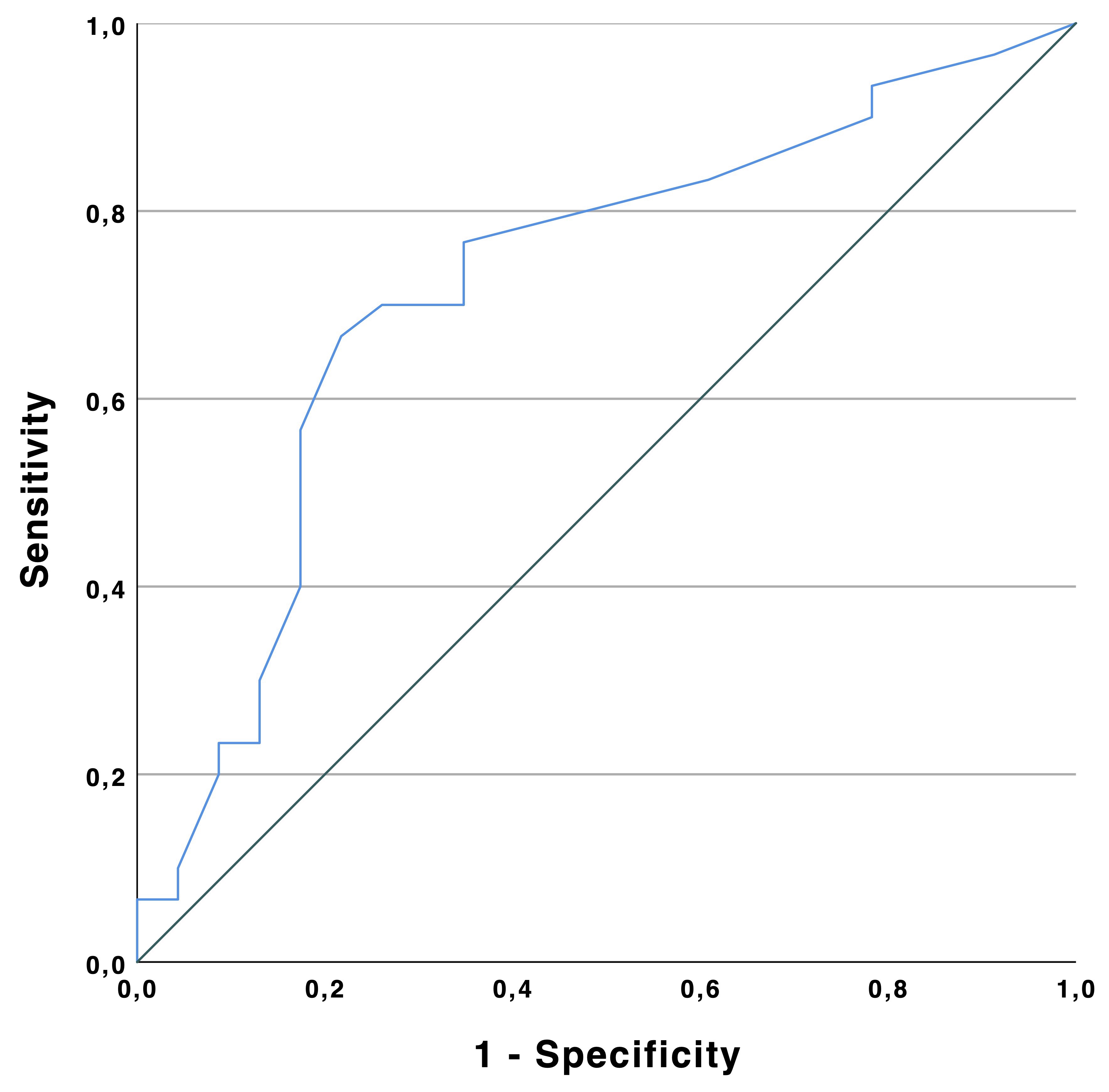

Supplement: Supplementary Figure S1 — ROC analysis of eGFR improvement and heart failure hospitalization. eGFR, estimated glomerular filtration rate. [file Image1.jpg]
